# Toward Sustainable Polyurethane Alternatives: A Review of the Synthesis, Applications, and Lifecycle of Non-Isocyanate Polyurethanes (NIPUs)

**DOI:** 10.3390/polym17101364

**Published:** 2025-05-16

**Authors:** Evangelia Balla, Dimitrios N. Bikiaris, Nikolaos Pardalis, Nikolaos D. Bikiaris

**Affiliations:** Laboratory of Polymer Chemistry and Technology, Department of Chemistry, Aristotle University of Thessaloniki, GR54124 Thessaloniki, Greece; euagelia226@gmail.com (E.B.); npardalis@chem.auth.gr (N.P.); nikospardalis99@gmail.com (N.D.B.)

**Keywords:** polyurethanes (PUs), non-isocyanate polyurethanes (NIPUs), polyhydroxyurethanes (PHUs), synthesis, applications, lifecycle, sustainability

## Abstract

In recent decades, scientific interest has increasingly focused on sustainable and green polymers. Within this context, considerable efforts have been devoted to the synthesis and exploration of eco-friendly non-isocyanate polyurethanes (NIPUs) as alternatives to conventional polyurethanes (PUs), solving the problem of isocyanate toxicity and other environmental problems that existed. This review article highlights the synthetic pathways of NIPUs and identifies the potential hazards associated with their production and end-of-life (EoL) stages. While in the literature there are several reviews regarding the synthesis of NIPUs, the current work distinguishes itself by providing a comprehensive summary of the latest research on NIPUs, with a particular focus on their lifecycle management, recyclability, and the challenges that hinder their scalability for industrial-level production. Advances in NIPU synthesis have made them strong candidates for a diverse range of applications. This review underscores the most notable examples of these advancements, emphasizing their potential to drive sustainable polymer development.

## 1. Introduction

Polyurethanes (PUs) are a remarkable class of polymers that, due to their outstanding mechanical, chemical, and physical properties, which include mechanical resistance, abrasion resistance, and chemical resistance, are used in a wide range of daily life applications. They possess durability comparable to metals, yet the elasticity of rubber makes them a versatile substitute for metals, plastics, and rubbers in numerous engineering and commodity applications. They were first introduced in 1947 by Otto Bayer and coworkers [1], a work driven by the necessity to address resource shortages and provide functional alternatives to polyamides, which were critical yet rare materials. At that time, PUs were used in military applications, including protective coatings for metals and aircraft fuel tank liners and insulating foams. In the years that followed, the utilization of polyether polyols at low cost for PU production skyrocketed the global use of flexible PU foams in many applications, such as automotive panels, air filters, and furnishing. Nowadays, PUs hold one of the largest market shares in non-flexible, high-resilience foam seating, rigid foam roofing insulation panels, surface coatings, high-performance adhesives, surface sealants, synthetic fibers, elastomeric wheels and tires, hard-plastic parts for electronics, and biomedical materials [2].

PUs are formed by the reaction of di/poly isocyanate and a diol or polyol, forming repeating urethane linkage in the presence of chain extender and other additives [3]. The main advantage of polyurethane consists of the fact that it is an extremely versatile family of polymers. Varying the polyol or the isocyanate may bring about a drastic change in the properties of the polyurethane; hence, polyurethanes can be manufactured in an extremely wide range of grades, in densities from 6 to 1220 kg/m^3^, and polymer stiffness from flexible elastomers to rigid and very hard plastics for high-performance applications [4,5]. What distinguishes polyurethanes from most thermoplastics is their unique production process. While thermoplastics are usually polymerized at large chemical factories and sold as granules or powders, polyurethanes are typically polymerized by the fabricator. Manufacturers provide the base materials or specially formulated chemical components for certain uses. This allows the fabricators to tailor the properties of PU to their specific needs [6]. Polyether polyols, for example, offer superior flexibility, while polyester polyols improve hardness and strength. Similarly, the choice of isocyanates, whether aromatic or aliphatic, plays a crucial role in the final properties of the polymer. This adaptability ensures that the potential applications of polyurethanes remain virtually limitless.

Referring to their classification, there are two main types of PUs, thermoplastic and thermosetting, each presenting different structures and functionalities. PUs of the thermosetting types are cured into cross-linked, three-dimensional networks and, hence, are not able to be reshaped or melted, which also results in an increase in their strength [7,8]. These PUs show exceptional heat and solvent resistance and increased strength, which means that they are suited for applications like coatings, adhesives, rigid or flexible foams, and sealants in the construction field [9,10,11]. In contrast, thermoplastic PUs comprise a linear polymer in the absence of intermolecular links between chains, which means they are able to melt and reform when subjected to proper temperature [12,13]. Thanks to their elasticity, flexibility, and abrasion and moisture resistance, they are most commonly used in cable sheathing, tubes, membranes, car parts, and shoes [14,15]. When deciding between thermosetting and thermoplastic PUs, the application focuses on certain characteristics, such as mechanical strength, flexibility and resistance to thermal and chemical degradation.

Within the circular economy concept and also considering the demand for limiting the environmental impact of plastics, a variety of methods have been developed for the recovery and recycling of PUs at the end-of-life stage [16,17,18]. Indeed, PU recycling remains an issue due to the complex cross-linked nature of foams and diverse formulations. Existing recycling practices like alcoholysis and glycolysis require a lot of energy, yield non-homogeneous output, and require the use of catalysts in order to prove effective [17,19]. Moreover, mechanical recycling, though simple, presents low-value addition, basically converting waste into fillers [18]. Biodegradation is another alternative, but it remains nascent and inefficient [20]. At the same time, the recrudescence of the environmental burden due to landfilling and incineration keeps pushing for advanced solutions. The development of novel recycling methods or bio-based alternatives is critical to reaching sustainability goals and reducing PU’s environmental impact.

Despite the advantages, and even though PU themselves are non-toxic materials, the manufacturing process relates to significant challenges. Firstly, side reactions that accompany the production of PU and also the involvement of phosgene, a highly toxic precursor for isocyanates, are major concerns. Phosgene poses risks during handling and production due to its acute toxicity and is responsible for health hazards as well as potential environmental release [21]. Moreover, isocyanate monomers, which are essential for the PU industry, have a highly reactive -N=C=O group, which also correlates with their toxicity [22]. Common monomers such as toluene diisocyanate (TDI), hexamethylene diisocyanate (HDI), and diphenylmethane diisocyanate (MDI) are categorized as carcinogenic, mutagenic, and reprotoxic (CMR) compounds [23,24]. They are inhaled or absorbed through the skin and have extensive health implications, which include occupational asthma, respiratory inflammation, and lung cancer [25]. The Bhopal disaster that took place in 1984 further highlighted the range of effects isocyanates have. The emission of methyl isocyanate (MIC) resulted in over 3000 deaths in the span of days and millions experienced adverse health effects in the long run [23]. This incident emphasized the importance of careful design of the regulations regarding the use of isocyanates in industrial operations. Monitoring and also constantly reducing the exposure of isocyanates is crucial to avoid such drastic consequences from occurring.

The various recycling technologies for material and chemical recycling have improved the recyclability of PUs in recent years. PUs are being subjected to mechanical recycling [26], chemical processing, thermochemical processing [27] and energy recovery [28] technologies. These recycling technologies open an effective, emerging and economic route for recycling thermoplastic and thermoset Pus, especially the ones in rigid foams and composite form [29]. Polyurethane foam has been successfully recycled using regrind technology in automotive seating [30]. Glycolysis of polyurethanes as a method of recycling can be economically acceptable, but still requires more development in order to endure more contamination in the post-consumer material. Existing technologies can reclaim the inherent energy value of polyurethanes and eliminate fossil fuel consumption [31]. Energy recovery is considered the only feasible disposal option for polyurethane waste at present when no market exists or can be developed for the recovered material. However, one of the major problems with polyurethanes, aside from their inherent toxicity, is the limited recyclability of the material. Waste-to-energy conversion and other advanced thermal processes such as gasification, pyrolysis, and two-stage combustion have made it possible to dispose of considerable quantities of scrap PU relatively efficiently [32,33,34]. While most of the feedstock recycling processes for plastics are technically promising, the practical recyclability of polyurethanes remains limited due to challenges such as low yield, high processing costs, and sensitivity to contamination. Further development is needed to overcome these recycling limitations of polyurethanes.

These challenges have created a demand for environment-friendly products to minimize the risk of hazards because of the highly toxic and reactive nature of isocyanates. This is where the non-isocyanate polyurethanes (NIPUs) emerge as new-generation polymeric materials that overcome the environmental and health problems associated with conventional PUs. To date, prominent works have been reported in the literature, establishing various ways to produce NIPUs that, unlike conventional PUs, are synthesized through non-isocyanate pathways, with the most common one being the reaction of cyclic carbonates and amines. This method produces urethane linkages without the use of toxic monomers [35,36]. Other advantages that increase the value of NIPUs are their reduced environmental impact, lower carbon footprint, and increased safety during production and application. They also exhibit characteristics, such as chemical resistance and thermal stability, while their production has utilized bio-based raw materials, following the principles of green chemistry [2,37]. Although NIPUs currently face challenges in commercialization due to slow polymerization, low molecular weight, and relatively low mechanical strength, advancements in amine–carbonate systems and innovative formulations are narrowing this gap. Recent innovations in catalyst systems and novel cyclic carbonate derivatives have demonstrated the potential for faster reactions and superior polymer properties. Promisingly, as NIPU technology continues to evolve, progress in material properties and manufacturing methods is anticipated, potentially enabling NIPUs to meet or even exceed the performance of conventional PUs [38,39].

In this review, the latest advancements in NIPU synthesis are summarized, mainly highlighting the bio-based routes, as well as the application of NIPUs in the fields of foams, coatings, adhesives, and biomedicals. In addition, their scaling-up potential, their environmental impact, as well as their recycling possibilities are summarized herein for the first time.

## 2. Toxicology and Health Impact Issues of Isocyanates

**Figure 1:**
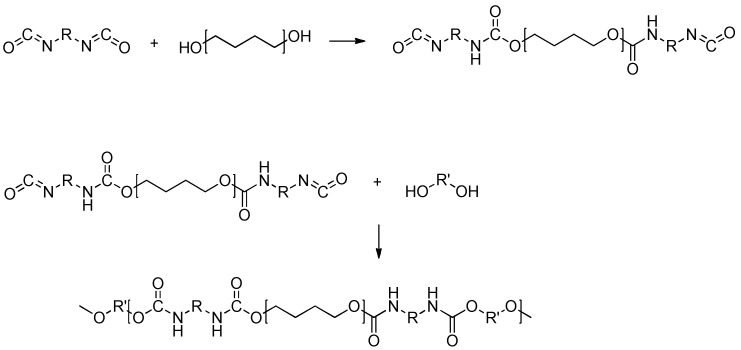
Synthetic pathways for (**top**) conventional polyurethane (PU) formation via reaction of diisocyanates with polyols and (**bottom**) non-isocyanate polyurethane (NIPU) formation via polyaddition of cyclic carbonates with diamines.

PUs are obtained through a two-step polyaddition reaction (Figure 1). Primarily, an aliphatic or aromatic diisocyanate reacts with a polyol, forming a linear polyurethane. This reaction is exothermic and takes place at low temperatures to prevent PU depolymerization that occurs at increased temperatures. Alternatively, when isocyanates are present in a twice or higher molar ratio of diols, the reaction produces low-molecular-weight oligomers. The oligomers can further undergo a second stage with diols or diamines, which are added as chain extenders, yielding traditional polyurethanes with high molecular weights. Reactions are carried out in the presence of a catalyst (i.e., 1,4-diazabicyclo[2]octane) to accelerate the reaction and sometimes a suitable solvent like dimethylformamide (DMF) or dimethyl sulfoxide (DMSO) is often employed, depending on the intended application [40]. During the formation of urethane bonds, isocyanate groups may react with water, leading to the formation of amines and carbon dioxide. While this reaction is beneficial in the synthesis of foams, it is undesirable when obtaining PU elastomers. The resulting amines can be further converted to urea moieties leading to hardened, unusable products. However, the most important class of PUs is thermosetting, which can be prepared in two approaches, like by incorporating multifunctional monomers, mostly polyols (-OH number greater than 3), or by using an excess of isocyanates. The latter method is mostly employed for elastomeric PU, where the secondary amine group of linear PU further reacts with the excess of isocyanates, creating cross-linked macromolecules.

Over 90% of commercially produced PUs are derived from aromatic polyisocyanates, which are favored over aliphatic and cycloaliphatic variants due to their higher reactivity with hydroxyl groups and the superior mechanical properties they impart to the resulting materials. Among aromatic isocyanates, TDI (toluene diisocyanate) and MDI (methylene diphenyl 4,4′-diisocyanate) dominate the industry, accounting for approximately 61.3% and 34.1% of the production, respectively. In contrast, aliphatic and cycloaliphatic polyisocyanates, such as hexamethylene diisocyanate (HDI) and isophorone diisocyanate, are used for applications like coatings, where resistance to discoloration from external sources is essential [41].

**Figure 2:**
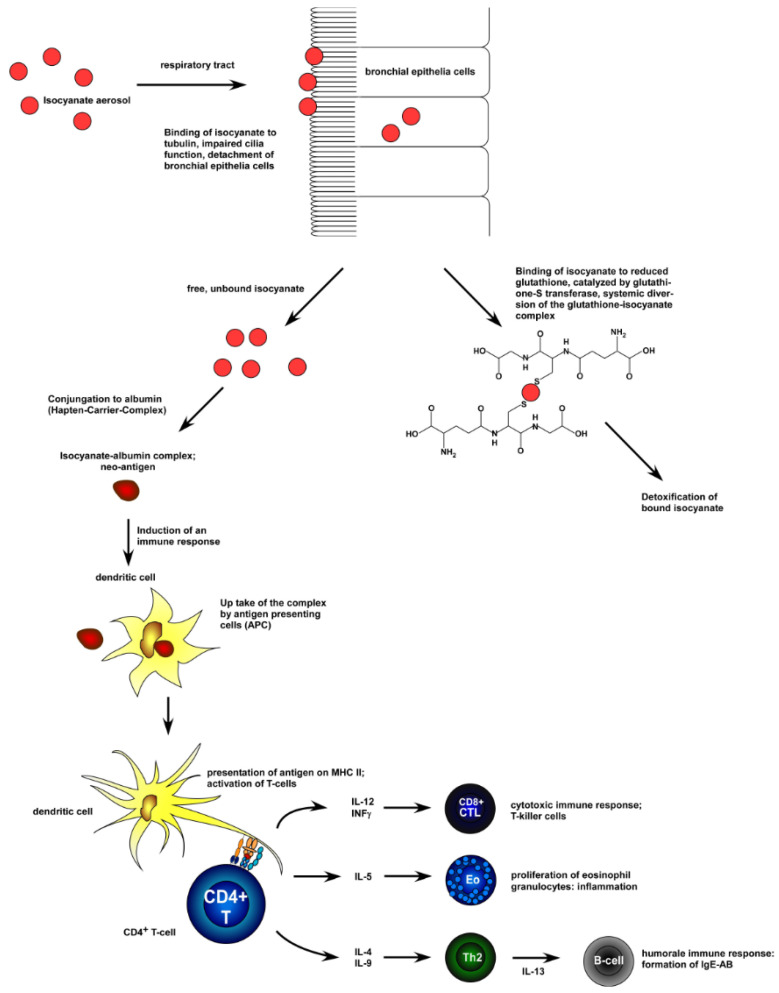
Predicted mechanism for the effects of isocyanate in the development of isocyanate-induced asthma [1].

According to European Parliament Regulation (EC No. 1272/2008), isocyanates are classified as extremely dangerous and poisonous compounds. As a result, isocyanates will gradually be prohibited in Europe under the Registration, Evaluation, Authorization and Restriction of Chemicals (REACH) rule. Many studies conducted in humans have addressed the inflammatory process underlying isocyanate-induced asthma danger, which is illustrated in Figure 2.

**Figure 3:**
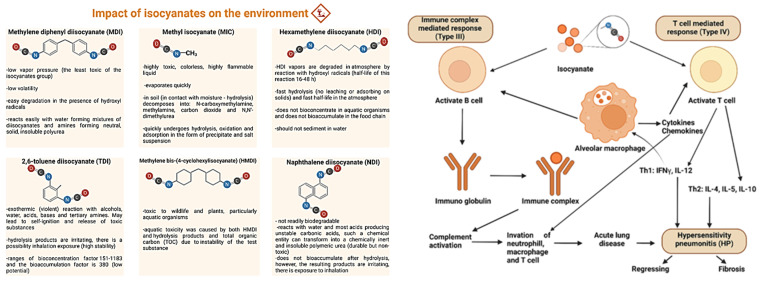
(**left**) Hazards of the most commonly used isocyanates and diisocyanates in PUs synthesis [23] and (**right**) immune system response to isocyanates [23,42].

Both isocyanates and isocyanides are classified in safety data sheets as harmful substances that can cause toxicity through inhalation, ingestion, and skin contact. However, due to their higher reactivity, extra oxygen atoms in their structure and the closely adjacent double bonds, isocyanates are much more toxic than isocyanides, and the primary routes to being poisoned by these compounds are dermal contact and inhalation. Specifically, inhalation of even a tiny amount may induce severe effects, such as asthma, respiratory inflammation and even lung cancer. Figure 3 (left) summarizes the most used isocyanates along with their characteristics and hazards. Owing to the above, the production and work with isocyanates must be strictly regulated and controlled [2]. The people facing the highest risk of harmful effects from exposure to isocyanates are workers and scientists who have daily contact with such substances. The response of the immune system to isocyanate exposure is described in Figure 3 (right).

Numerous industries, such as the chemical industry, the automotive industry, the woodworking industry, and the foundry industry are workplaces where exposure control and regular testing are of great importance. Replacing isocyanates will offer indispensable health benefits but necessitates a thorough evaluation of alternative methods to avoid unintended exposures.

The various types of polyurethane waste products, consisting of either old recycled parts or production waste, are generally reduced to a more usable form, such as flakes, powder or pellets, depending on the particular type of polyurethane that is being recycled. Due to the variety of physical, chemical, thermal and mechanical properties, PUs have a broad range of applications such as automobile seating, furniture, carpets, refrigerators, insulation boards, adhesives, and medical.

## 3. Investigation of Potential NIPU Toxicity

The growing significance of sustainability within the chemical industry has led to increased awareness regarding safety and health standards. Ecotoxicity has become an important evaluation criterion that should be considered in the development of novel bio-based monomers.

**Figure 4:**
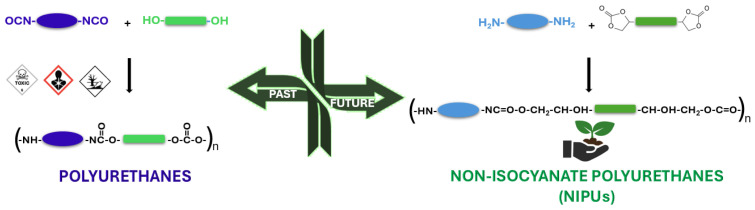
Comparative synthetic reactions for conventional PU vs. NIPU formation.

Despite the growing interest and progress in NIPUs as safer alternatives to traditional PUs, there is a notable lack of published studies specifically addressing the toxicity of NIPUs themselves. In the absence of such data, it is crucial to evaluate the toxicity profiles of the monomers used in their synthesis, such as cyclic carbonates and bio-based diamines. Understanding the potential health and environmental impacts of these components and their production is essential to ensure that NIPUs fulfill their promise of being a safer and more sustainable alternative while maintaining performance standards (Figure 4).

Dialkyl carbonates are important organic compounds and chemical intermediates and are considered more sustainable alternatives due to their comparably low toxicity, decrease in irritating or mutagenic effects, and biodegradability for important representatives. Owing to these features, they have gained interest as alternatives for toxic and carcinogenic chemicals used during the production of isocyanate PUs, most prominently phosgene. Among linear carbonates, the most important ones are dimethyl carbonate (DMC) and diethylcarbonate (DEC). Mundo et al. [43] compared several routes usually employed for the synthesis of the most common cyclic carbonates such as DMC and DEC. They highlighted that the reaction between epoxides and CO_2_ is an efficient route to yield cyclic carbonates, also at the industrial level. This reaction offers numerous advantages from a green chemistry perspective since it uses CO_2_ as a renewable and non-toxic monomer. Additionally, the reaction can take place under solvent-free conditions.

Despite the chemical similarities one can find between traditional PUs and NIPUs, the direct replacement of conventional PUs with NIPUs has been proven to be challenging in practice, and new developments are required [44]. In terms of toxicity, the critical advantages of NIPUs over traditional PUs are their ability to avoid the use of diisocyanate substrates and the use of renewable resources as feedstocks for NIPU synthesis. These feedstocks, mostly from naturally occurring compounds with unsaturated bonds, can be epoxidized and converted into glycols to react with isocyanates for the production of PU or used in CO_2_ cycloaddition to form cyclic carbonates, which react with amines to create NIPUs. Of these two pathways, the latter represents a much greener pathway; it avoids toxic intermediates and contributes toward a reduction in CO_2_ emissions [45].

Remarkably, although NIPUs indicate a safe profile, as they eliminate toxic isocyanates, they have not been widely applied at an industry level. As a consequence, the literature lacks long-term toxicological studies in order to comprehensively investigate their safety, especially under continuous exposure and in different applications.

## 4. Synthetic Routes of NIPUs

Dyer and Scott introduced a method for the synthesis of polyurethanes with no involvement of isocyanates in 1957 [46]. In this study, the authors proposed the synthesis of urethanes and cyclic urea by utilizing ethylene carbonate and various diamines to yield b-hydroxycarbamate. These compounds were then subjected to polycondensation via a transesterification reaction. This pioneering work established the foundation for the development of NIPUs and emerged as one of the most prominent topics of research. To date, there are four approaches to synthesizing NIPUs, including polycondensation, ring-opening polymerization (ROP), rearrangement reactions, and polyaddition of cyclic carbonates and amines.

### 4.1. Polycondensation Reactions (Transurethanization Pathway)

**Figure 5:**
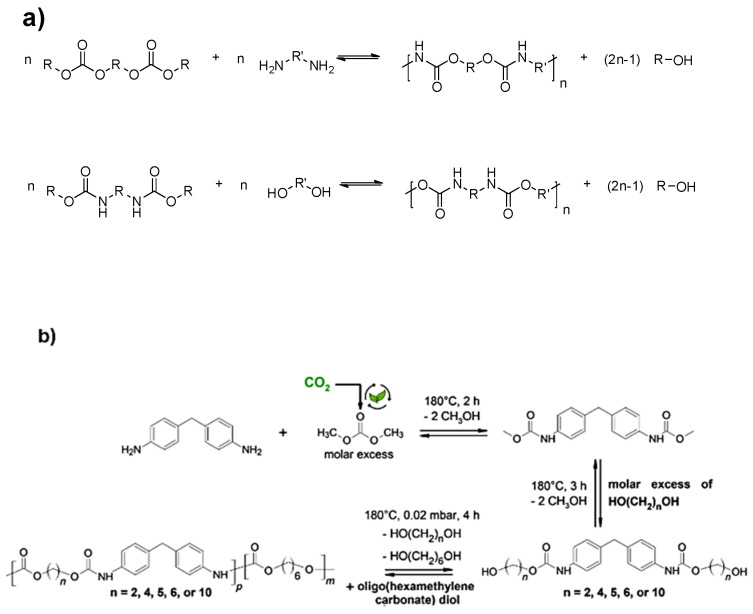
(**a**) Schematic representation of the polycondensation route in NIPU synthesis, and (**b**) the transurethane polycondensation reaction between BHAC and OCD yielding NIPCU differing in the structure of hard segments [3].

The polycondensation method in NIPU synthesis mainly involves the reaction between linear activated carbamates and diols (transurethanization) or the reaction between linear activated dicarbonates and diamines as described in Figure 5a. Carbamates used for this method are derived from phosgene or its intermediates (isocyanates and chloroformates), indicating that this approach is as toxic as the conventional one [47,48]. In recent years, some sustainable alternatives for carbamate production have been proposed, such as the reaction of alcohols with urea or its derivatives. This method avoids the use of phosgene entirely by utilizing urea as the carbonyl source to form the carbamate linkage. Qin et al. [49] investigated the synthesis of phenyl methyl carbamates through the reaction of aniline, urea, and methanol using various kinds of catalysts.

Carbamates have also been prepared in good yields through the reaction of amines, alcohols, carbon monoxide, and oxygen in the presence of novel metal catalysts. Shi et al. [50] reported an efficient and clean synthesis of carbamates through oxidative carbonylation of aromatic amines using polymer-immobilized gold catalysts. Except for carbon monoxide, carbon dioxide has also been frequently used, in various conditions and forms, as a cheap and safe alternative for the synthesis of carbamates. Peterson et al. [51] studied the synthesis of carbamates through the reaction of secondary amines with primary and secondary alcohols using gaseous CO_2_ through a Mitsunobu reaction employing DBU and a DBAD/Bu3P system. Wołosz et al. [52] presented the synthetic procedure of aliphatic–aromatic non-isocyanate poly(carbonate–urethanes) (NIPCUs) via transurethanization reactions based on dimethyl carbonate as a green substitute for phosgene in the presence of CO_2_ (Figure 5b). Thus, the polycondensation route involves the synthesis of NIPUs through a greener perspective in comparison with the symbiotic route for PU synthesis but still lacks in terms of “non-toxicity”, as the carbamates used for this method are derived from phosgene or its intermediates.

### 4.2. Ring-Opening Polymerization of Cyclic Carbamates

**Figure 6:**
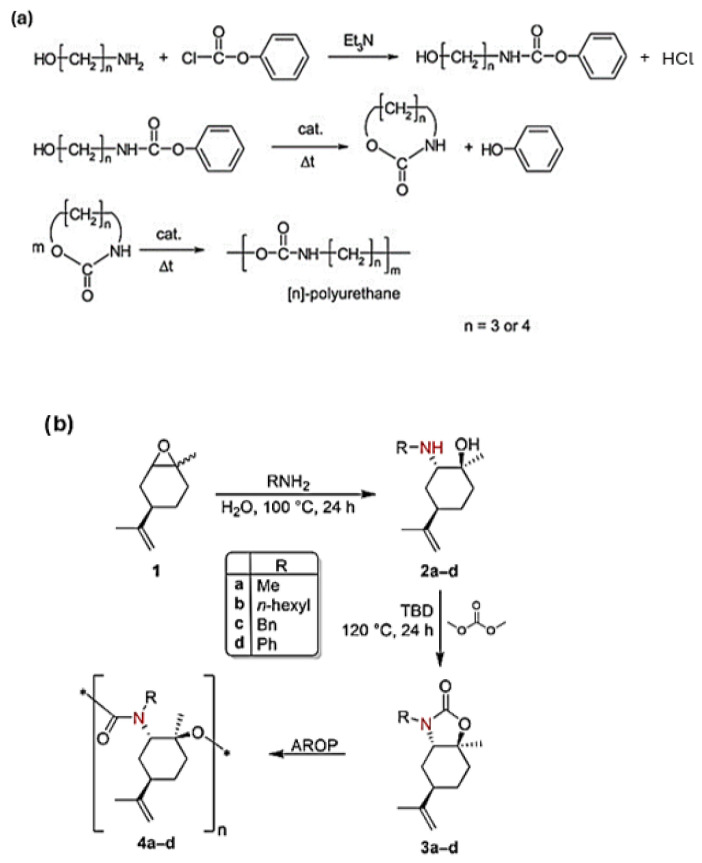
(**a**) NIPU synthesis through polymerization of cyclic urethane [4] and (**b**) reaction pathway for the synthesis of NIPU from (R)-(+)-limonene oxide [5].

Another way of producing NIPUs suggests the ring-opening polymerization (ROP) of 6–7-membered cyclic carbamates [53]. Although these reactions proceed without the release of byproducts, they are characterized as temperature-governed, which means that high temperatures are required to produce effective materials. At the same time, cyclic carbamate preparation involves the use of phosgene or its derivatives, increasing the toxicity to a relatively high level [54,55]. For example, it is notably reported in the literature that the reaction of 3-aminopropan-1-ol or 4-aminobutan-1-ol with phenyl chloroformate yields an aliphatic–aromatic urethane, which subsequently converts into a cyclic monomer that distills from the reaction mixture under reduced pressure. This cyclic monomer is then converted into [n]-polyurethane, an NIPU with an alkane bone of 3 or 4 carbon atoms, via ROP. However, as mentioned above, phosgene is used in the initial preparation of phenyl chloroformate (Figure 6a) [56]. Hence, the ROP of cyclic carbamates has been scarcely investigated. Recently, greener methods for cyclic carbamate production have been introduced [57]. Rubino et al. [58] suggested the preparation of NIPUs by ROP of a cyclic carbamate derived from (R)-(+)-limonene oxide (Figure 6b).

### 4.3. Rearrangement Reactions

**Figure 7:**
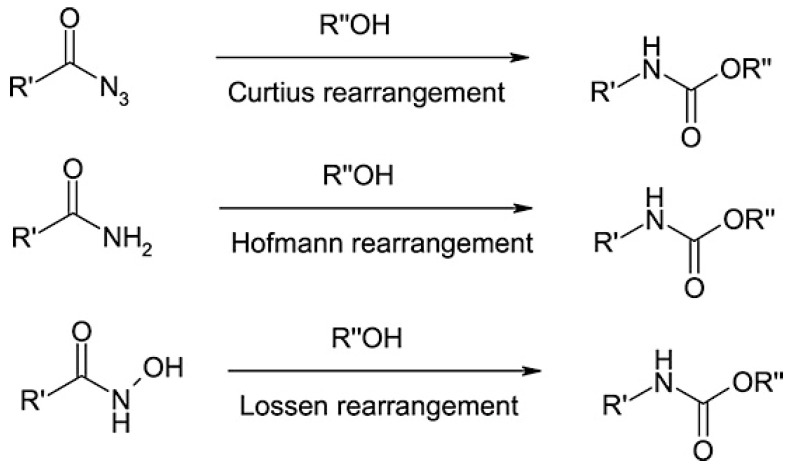
Schematic representation of rearrangement NIPU synthesis reaction [21].

Among the methods for producing NIPUs, one can find the rearrangement reaction including various types such as Curtius [59], Hofmann, or Lossen rearrangements (Figure 7) [60].

Most of these reactions use toxic reactants such as acyl azides, carboxamides, and hydroxamic azides. Therefore, they utilize isocyanate compounds, even if they are generated in situ at that moment, and so these methods are still not less toxic than the conventional one. Additionally, halogens such as bromine and chlorine are used in the production process, resulting in further environmental burden due to the creation of toxic waste. To overcome these, Kreye et al. [61] described an environmentally benign approach of Lossen rearrangement with dimethyl carbonate (DMC) as a green activation reagent of hydroxamic acids in the presence of catalytic amounts of tertiary amines and small quantities of methanol.

### 4.4. Polyaddition of Cyclic Carbonates and Amines

**Figure 8:**
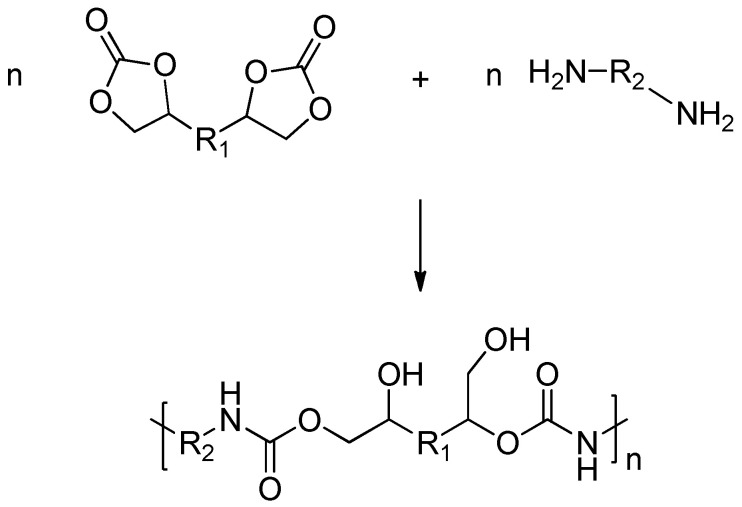
Schematic representation of polyaddition reaction.

This reaction consists of a two-step process: first, the formation of a cyclic carbonate oligomer and, second, a reaction with amines or polyamines leading to the final NIPU (Figure 8). Polyaddition is considered the optimal method for synthesizing NIPUs since it does not require phosgene to produce cyclic carbonates, unlike other monomers typically used in NIPU synthesis. Cyclic carbonates show low toxicity and are user-friendly, as they are non-sensitive to moisture. Similarly, the polyamines used for ring opening are also low in toxicity. As an additional advantage, a wide range of bio-derived or biodegradable compounds can easily be converted into corresponding monomers (both cyclic carbonates and amines) suitable for participating in these reactions [39,62].

With the formation of every urethane linkage, a primary or secondary hydroxyl group is also formed, hence, these NIPUs may be alternatively named polyhydroxy-urethanes (PHUs). These hydroxyl groups are capable of forming intermolecular hydrogen bonds with the urethane group [63]. The intramolecular hydrogen bond blocks the carbonyl carbon, resulting in low susceptibility of the whole urethane group to hydrolysis and, hence, improved hydrolytic stability, well above that of conventional Pus, could be achieved. Moreover, materials containing intramolecular hydrogen bonds display chemical resistance 1.5–2 times greater than materials having similar chemical structure without such bonds [64].

The two main disadvantages identified in the polyaddition method are, firstly, the generally low reactivity between carbonyl groups in cyclic compounds and amines, and second, the limited reaction yield during polymerization at room temperature, thus leading to low-molecular-weight PHUs [35]. Overall, the selection of the individual components plays a crucial role in determining the properties of NIPU, and careful consideration of their source and ratio is necessary to tailor the material properties and meet specific application requirements.

## 5. Bio-Based Synthesis of NIPU

### 5.1. Bio-Based Synthetic Routes for Cyclic Carbonates

**Figure 9:**
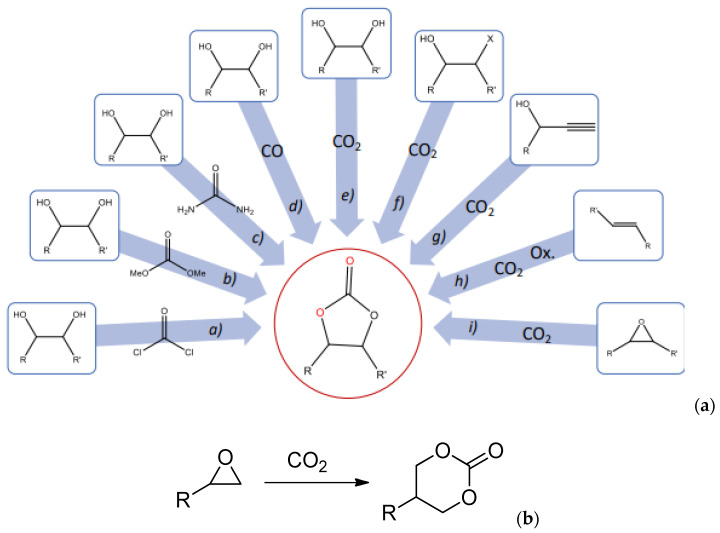
(**a**) Routes toward the production of cyclic carbonates, excluding inorganics [6] and (**b**) 5-membered cyclic carbonate formation by carbonation of oxirane ring [70].

Through the years, several methods of forming cyclic carbonates (especially 5-membered) have been proposed [65]. Figure 9a summarizes some common routes to prepare cyclic carbonates using different starting materials. The most commonly studied and sustainably attractive route remains the carbonation of oxiranes in the presence of a catalyst (Figure 9b) [66]. This reaction is favored since it utilizes CO_2_, a renewable, non-toxic, and abundant reactant, besides being relatively facile and straightforward. Aiming to improve reaction efficiency, recent research has investigated a variety of catalysts to increase the electrophilicity of the cyclic carbonate group or the nucleophilicity of the amine group. Metal-free systems, such as tetrabutylammonium bromide (TBAB), have shown great effectiveness compared to organometallic catalysts or other quaternary ammonium salts in terms of promoting the opening of the oxirane ring and the fixation of CO_2_ [67,68,69].

The synthesis of cyclic carbonates primarily used propylene carbonate and ethylene carbonate, obtained from propylene oxide and ethylene oxide, respectively [46,71], that derive from petroleum-based chemicals.

In recent years, the growing emphasis on compliance with green chemistry principles is moving towards bio-based and renewable feedstocks for the preparation of epoxides. Widely studied epoxides, such as epichlorohydrin and glycidol, are prepared from glycerol, a major byproduct of biodiesel production on a large scale [72]. Tanaka et al. [73] developed a well-defined PS-supported alkylammonium salt catalyst, which showed excellent catalytic activity and reusability for transesterification of methyl esters with glycidol, giving glycidyl esters in high yields. Additionally, growing attention has been devoted to epoxides derived from precursors such as vegetable oils [38,74,75], lignin derivatives, terpenes [76,77], vanillin [78,79], tannin [80], glycerol, and isosorbide [81].

Triglycerides are a renewable and promising feedstock for NIPU synthesis, offering wide structural and functional versatility, being an excellent alternative to fossil-based resources owing to their biodegradability, non-toxicity, low cost, and inherent functionality. Vegetable oils, rich in active triglyceride-moieties, are suitable for chemical modification, as they can be easily converted into epoxidized derivatives and, subsequently, into poly(cyclic carbonates) through CO_2_ reactions, enabling the production of PHUs [82]. Extensive research has explored edible oils, including sunflower, soybean, linseed, and palm oil [69,82,83,84], as well as non-edible sources [6], such as rubber seed oil and cardanol. For instance, Tamami et al. [69] demonstrated the conversion of epoxidized soybean oil into carbonated derivatives and, subsequently, the synthesis of PHUs. Other similar studies have employed sunflower oil and rosin-derived cyclic carbonates for developing NIPUs [6,69,82,83,84,85,86]. Pouladi et al. [75] explained the synthesis of a bio-resourced resin via epoxidation and carbonation of linseed oil in acidic conditions under the purging of CO_2_. The resins were cured with diethylenetriamine to form NIPU networks. Recently, Werlinger et al. [87] employed used cooking oils derived from olive and sunflower for the synthesis of new bio-derived cyclic carbonates catalyzed with metal-free bifunctional organic compounds under mild and solvent-free reaction conditions. Once cyclic carbonates were synthesized, the design of NIPUs with different structured diamines was successfully carried out.

Lignin-based bisphenols are considered also valuable green alternatives for the synthesis of cyclic carbonates, since they offer an aromatic structure and hydroxyl functionalities that make them suitable for NIPU precursors [88]. For example, Chen et al. [89] synthesized a bis(cyclic carbonate) from creosol-based bisphenol via glycidylation with CO_2_ cycloaddition, followed by polyaddition with diamines. Similarly, Janvier et al. [90] utilized syringaresinol to synthesize thermoplastic PHUs with high Tg values of 63–98 °C. Other bio-based precursors include vanillin and tannic acid, the latter being used by Esmaeili et al. [91] to produce PHU networks through CO_2_ fixation. Derived from lignocellulosic biomass ferulic acid and furan-2,5-dicarboxylic acid (FDCA), they have both been explored for synthesizing thermoset and thermoplastic PHUs through efficient catalytic systems.

Polyols like pentaerythritol and trimethylolpropane can be enzymatically derived from starch and further hydrogenated. Fidalgo et al. [92] converted D-mannitol into bifunctional carbamates via transesterification, followed by ring-opening polymerization with diamines. Similarly, Mazurek-Budzynska et al. [93] synthesized cyclic carbonates from sugar polyols like sorbitol using dimethyl carbonate (DMC) and potassium carbonate, enabling the production of cross-linked PHUs. Furtwengler and Avérous [94] developed a solvent-free process to synthesize bis(cyclic carbonate) from sorbitol under mild conditions, achieving 50% conversion. Aouf et al. [95] utilized natural phenolic compounds such as gallic acid for bio-based epoxy groups, which were subsequently cured with diamines into NIPU materials. Schmidt et al. [96] proposed a green route to synthesize sorbitol tricarbonate from glycerol byproducts, showcasing its potential as a precursor for NIPU production.

Terpenes, being highly unsaturated and ester-free, are abundant and offer unique potential as precursors for NIPU synthesis. Bähr et al. [97] developed a terpene-based route using cyclic limonene dicarbonate. Under 30 bar pressure and 140 °C, with TBAB as a catalyst, 34.4% CO_2_ was incorporated into limonene dioxide. The resulting cyclic carbonates were then cross-linked with various diamines to produce PHUs. Bähr et al. reported a versatile new route to linear, as well as cross-linked NIPUs derived from the novel cyclic limonene decarbonate, based on (R)-limonene, a terpene extracted from abundant citrus peels waste from juice production [97].

Cashew nutshell liquid (CNSL), a non-edible byproduct of the cashew shell industry, holds great potential for producing sustainable materials like NIPUs due to the phenolic nature and unsaturated aliphatic chain of its main component, cardanol. Kathalewar et al. [98] synthesized cyclic carbonate from CNSL via CO_2_ addition to epoxidized cardanol at 120 °C under elevated CO_2_ pressure using TBAB as a catalyst. These cyclic carbonates were further reacted with diamines in the presence of triethylamine. Similarly, Dworakowska et al. [99] developed bio-based epoxy foams using epoxidized cardanol and fatty acid-based diamines.

### 5.2. Bio-Based Synthetic Routes for Polyamines

The mechanism of NIPU synthesis, through the polyaddition route, is explained by a simultaneous nucleophilic attack of an amine over a cyclic carbonate, followed by a deprotonation reaction. In the first stage, the nucleophilic attack of the amine at the carboxyl group results in the formation of a tetrahedral intermediate. In the second stage, the latter is protonized through an attack of another amine, resulting in the removal of hydrogen ions. Afterwards, favored by the strong electron-withdrawing effect of nitrogen atoms, the carbon–oxygen bond breaks and the new generated alkyl–oxygen ion combines with hydrogen ions, resulting in a rapid formation of the product [100]. The literature indicates that higher reaction temperatures yield greater efficiency.

The properties of NIPU can be tailored based on the amine used during the previously discussed stage. The selection of amine can play a crucial role in the molecular weight, cross-link density, and branching degree, which, in turn, dictates the mechanical and thermal properties of the resulting polymer. Owing to higher nucleophilicity, primary amines are more reactive than secondary or tertiary amines. Diamines usually produce linear polymers, while polyamines can lead to branched or cross-linked networks [101]. Moreover, the employment of aliphatic diamines typically enhances the swelling resistance, in contrast to aromatic diamines, which can improve the chemical, mechanical, and thermal stability of the resulting polymer [102]. For instance, the incorporation of hexamethylene diamine showed a decrease in glass transition temperature (Tg) from 73 °C to 51 °C, indicating enhanced flexibility [103]. Additionally, aromatic diamines like 1,2-di(p-aminophenoxy)ethane improved thermal and mechanical properties, as evidenced by increased Young’s modulus [104].

Currently, academic research is focused on the development of bio-based amines for NIPU production in order to reduce reliance on fossil fuels and mitigate the environmental impact associated with conventional amine production methods [103]. Using catalytic reductive amination, various carbohydrate-derived substrates can be converted into a wide range of alkyl polyamines, alkanolamines, and aliphatic heterocyclic amines that are non-toxic and safe to handle. Froidevaux et al. suggested the synthesis of amines from all sorts of biomass such as chitosan, terpenes, vegetable oil derivatives, sugar derivatives, lignin, and proteins [105].

A great variety of bio-based sources are reported in the literature as starting materials for polyamines’ synthesis [101]. Among them, amino acids are considered renewable materials that can be used to produce precursors for the synthesis of polyamines because of the presence of the nitrogen atom in their structure. Decarboxylation of amino acids can lead to formation of either mono- or bifunctionalized amines, depending on the structure of the starting molecules [106]. Lipid-based molecules are reported to be successfully converted into diamines or polyamines. Biermann et al. [107] explored different paths aiming to functionalize unsaturated fatty acids. Among the various techniques, one can find, for instance, the epoxidized triglycerides, which can be converted into azide derivatives in the presence of ionic liquid as a catalyst [92]. Similarly, Zhao et al. [86] proposed a promising strategy that involves implementing a multistep process in order to synthesize secondary amines from epoxidized triglycerides. Vegetable oils can be functionalized into amines via a thiol–ene (thiol and alkene) coupling reaction [85]. Stemmelen et al. [85] took advantage of this method and demonstrated that unsaturated grapeseed oil can result to the synthesis of a polyamine through a reaction of cysteamine hydrochloride and UV-initiated thiol–ene chemistry. In this context, Turunc et al. [101] synthesized a polyamine combining the cysteamine hydrochloride with grapeseed oil.

Bio-based dicarboxylic acids such as succinic, adipic, and azelaic acids are attracting great research interest due to their broad use as a precursor to synthesize valuable chemical materials [101,108,109]. Lastly, polyamines can be synthesized via catalytic amination of bio-based alcohols. Ethylene glycol, for instance, can be obtained from cellulose and can be exploited as a potential precursor for amination reaction [109]. Isosorbide, which derives from sorbitol, can be converted into corresponding mono- and diamines via the introduction of nitrile compounds, followed by catalytic hydrogenation [110].

In summary, among the aforementioned methods, which are available for the synthesis of NIPUs, the polyaddition of cyclic carbonates with amines appears to be the most promising and innovative. This synthetic route not only aligns well with green chemistry principles by avoiding the use of toxic isocyanates but also offers wide versatility in terms of reaction conditions and choice of reactants. This sustained attention it has gained highlights its potential for scalable, sustainable applications and its relevance in advancing eco-friendly polymer production.

## 6. Circular Economy in NIPUs: A Lifecycle Perspective

**Figure 10:**
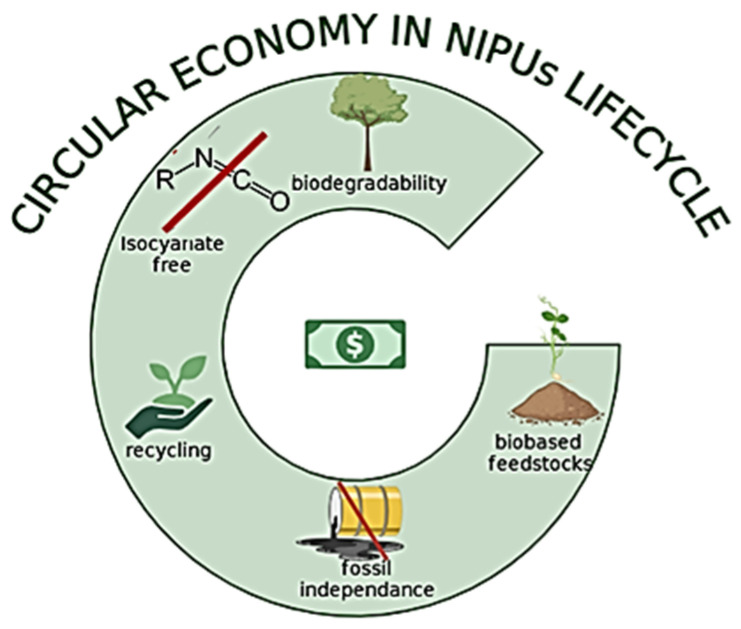
Circularity in NIPUs’ economy lifecycle.

Aiming to enhance the PUs’ sustainability, it is, firstly, essential to advance technologies for producing these materials from bio-based feedstocks; secondly, to expand methods for recycling PU monomers; and, thirdly, to explore synthetic routes that sidestep the use of isocyanates. The substitution of fossil-based PUs with NIPUs from bio-based chemical building blocks can potentially minimize their environmental impact and enhance their sustainability (Figure 10).

Nonetheless, the bio-based content is simply one of the twelve foundational principles of green chemistry. Although promising for de-fossilizing PU production, the use of bio-based building blocks will still have various environmental impacts. Biomass extraction and its alterations must enable the employment of green solvents and catalysts, as well as low energy consumption without loss in the atom economy [111]. The intent for the end of life (EoL) should be always considered during the development of new products such as bio-based PUs, e.g., designing for recycling/biodegradation, or recovery of initial monomers, to enable the move toward circular economy principles.

## 7. Sustainability and Environmental Impacts

**Figure 11:**
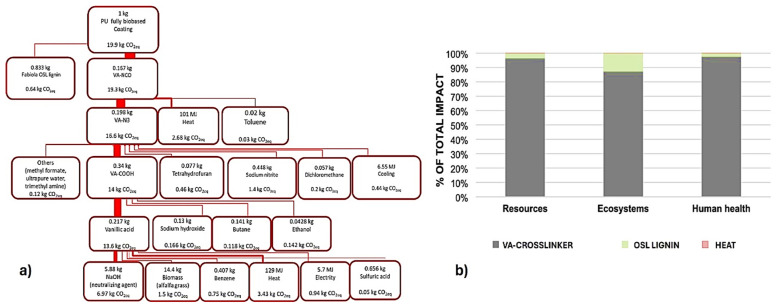
(**a**) Most significant contributors to global warming potential (GWP) impacts, i.e., CO_2_ eq emissions, for the production of 1 kg of fully bio-based PU coating made from OSL and VA cross-linker. Economic allocation was applied to the products of the biorefinery process for the production of VA and (**b**) relative impacts associated with OSL and VA cross-linker in the production of 1 kg of bio-based PU coating for different endpoint impact categories [7].

Currently, there are scarce data from published studies regarding the lifecycle scenarios of bio-based PUs and their associated environmental impacts. In one of these studies, Wray et al. [112] conducted an LCA to evaluate the carbon footprint (CO_2_ eq) of producing PU coatings using bio-based and hybrid materials. The scientific group compared an entirely bio-based coating produced by organosolv lignin (OSL) and a bio-based cross-linker from vanillic acid (VA) with hybrid coatings, which combined a fossil-based cross-linker with either OSL or partially depolymerized OSL (PDR-OSL). The fully bio-based PU coating generated 19.9 kg CO_2_ eq per kg (Figure 11a), which was 92% and 87% higher than the OSL and PDR-OSL hybrid coatings, respectively, due to the complex production method of the VA cross-linker. Additionally, when comparing bio-based and hybrid coatings with conventional water-borne PU (WBPU) coatings, hybrid coatings with OSL demonstrated similar or lower greenhouse gas emissions. These findings indicated that incorporating lignin as a bio-based polyol could achieve emission levels comparable to commercially available fossil-based coatings. Impacts related to damage to ecosystems, human health, and resource scarcity from the production of fully bio-based coatings indicate that most (average 96%) of the impacts are attributed to the production of the VA cross-linker. Considering that the bio-based coating consists of 80% lignin by mass, this underscores the relatively high environmental impacts of the bio-based VA cross-linker across all impact categories (Figure 11a). However, these results are not entirely restrictive, since bio-based cross-linkers using dissimilar bio-based materials and different routes of production can be considered for PU coating production.

**Figure 12:**
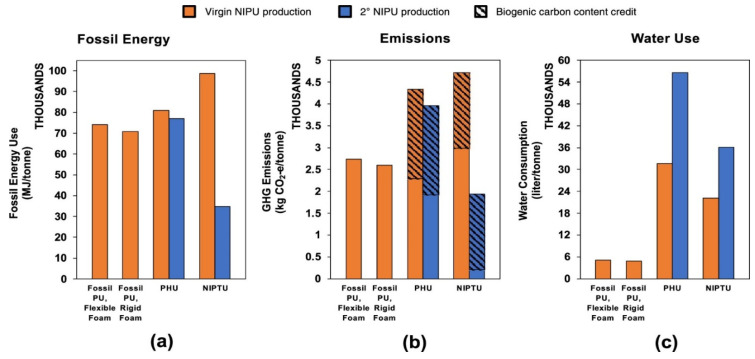
Lifecycle impacts: (**a**) fossil energy consumption, (**b**) GHG emissions, and (**c**) water use impacts of NIPU production and reprocessing. Impacts for PHU and NIPTU production are compared to the production of flexible and rigid PU foams [113].

In another research study, Liang et al. [113] compared conventional isocyanate-based PUs to poly(hydroxy urethanes) (PHUs) and non-isocyanate polythiourethanes (NIPTUs). Figure 12 summarizes the data obtained from the work. It was found that PHU production requires 80,900 MJ per ton, which is 12% more fossil energy than fossil-based PU foams, while NIPTU production requires 98,600 MJ per ton, 35% more than fossil-based PU foams, respectively. They also studied the depolymerization and polycondensation reactions for the reprocessing of NIPUs into secondary (2°) NIPUs. Reprocessed NIPU from PHU requires 4.7% less fossil energy compared to producing new PHU, while reprocessed NIPU from NIPTU requires 64.8% less fossil energy than producing new NIPTU. Water consumption for PHU production (31,600 L per ton) is six times higher than that for fossil-based PU foam production, respectively, while water consumption for NIPTU production (22,100 L per ton) was four times greater. All in all, it was concluded that contrary to fossil energy use and emissions, reprocessing NIPU requires significantly more water than producing virgin NIPTU.

## 8. End of Life of PUs and NIPUs

The average recycling rate of PUs in 2015 in the U.S. was calculated at 5.5%, less than any other plastic. This significantly low rate is mainly attributed to the challenge and high costs associated with collection and extraction from products such as car seats and shoes. Because of the diversity in forms and applications, a general “single recycling pathway” for PUs is difficult to develop [114]. To address this, several methodologies have evolved to address the problems associated with PU wastes [115]. PU coatings in particular are very difficult to recycle given that they are typically mixed (and disposed of) with a variety of other materials, such as metals or wood, making their separation and recovery quite challenging.

Waste management is usually carried out by landfilling or incineration, in most cases without energy recovery. Disposal via landfilling and incineration of PU waste can be modeled, but no distinction can be made between bio-based, hybrid, and fossil-based PU coatings. It is reasonable to assume that the impacts of landfilling or incineration of bio-based or hybrid coatings would be different than those of fossil-based coatings. As part of the same study, Wray et al. [112] modeled the end-of-life pathways of incineration and landfilling for 1 kg of fossil-based PU waste. The end-of-life impacts of fossil-based PU coatings, including incineration and landfilling, were significant, with incineration contributing to 3.51 kg CO_2_eq/kg of coating and landfilling contributing to 0.004 kg CO_2_eq/kg. Energy recovery during incineration can reduce the impact by up to 55%. When combined with production impacts, end-of-life scenarios can account for 1% to 79% of GWP impacts for fossil-based PU coatings, suggesting that bio-based or hybrid coatings could also have substantial end-of-life impacts, particularly if incinerated.

Physical recycling is successfully used with thermoplastic polymers but, unfortunately, it is useless for thermoset PUs due to their thermostability. The cost-effective simple mechanical method involves grinding or pressing PU wastes for reuse; however, it exhibits poor mechanical properties of the recycled products [33]. On the other hand, chemical recycling route involves glycolysis, hydrolysis, and aminolysis to recover valuable raw materials such as polyols for use in the synthesis of new PUs. This route may be plausible but would be linked with environmental impacts from the use of numerous chemicals. Presently, glycolysis is the most extensively used chemical recycling process for PUs. It involves a transesterification reaction in which the ester group joined to the carbonyl carbon of the urethane is interchanged by the hydroxyl group of a glycol. At industrial level, glycolysis processes have not yet been established, owing to the excess and high cost of the cleavage agent required to ensure a proper phase separation. Few companies at the moment, such as BASF, Getzner Company, and Troy Polymers, hold patents for the development of single-phase glycolysis industrial processes of PUs (U.S. Patent No. 5,556,889, U.S. Patent No. 6020386, and U.S. Patent No. 6,750,260) [17,116,117,118]. Thermo-chemical techniques involving pyrolysis and gasification may ensure energy recovery along with chemical feedstocks but raise concerns about environmental emissions [28].

**Figure 13:**
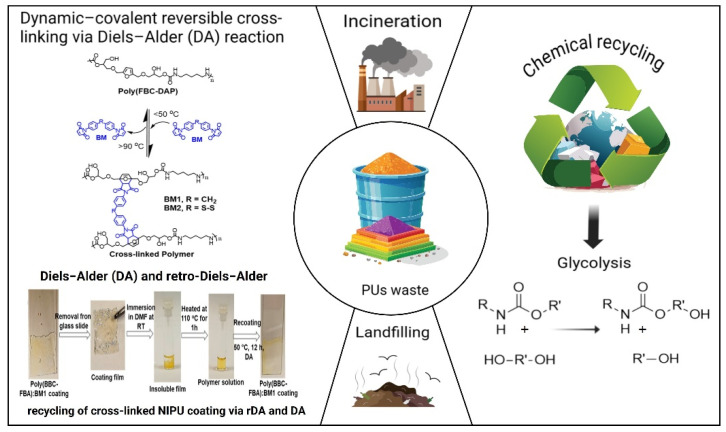
Overview of end of life of PU waste.

Choong et al. [119] prepared bio-based NIPUs containing dynamic-covalent reversible cross-linking via furan–maleimide Diels–Alder (DA) reaction that were both recyclable and healable via three different mechanisms. After heating at 110 °C for 1 h, the cross-linked NIPU coating film produced polymer solution in DMF. The polymer solution was then applied to recoat the substrate several times, thus proving its chemical reversibility. Still on a research scale, the group of Li et al. [120] synthesized NIPU from renewable bis(6-membered cyclic carbonates) (iEbcc) and amines. The resulting NIPU possessed great mechanical performance and thermal stability, and, at the same time, it could be remolded via transcarbamoylation reactions. In addition, recyclable NIPU could be chemically degraded into bi(1,3-diol) precursors with high purity (>99%) and yield (>90%) through alcoholysis, and degraded products could be used to reproduce NIPUs with similar structures and properties as the first samples. Figure 13 describes the end-of-life disposable options of PU waste including the previously mentioned DA reaction, chemical recycling, incineration, and landfill disposal.

To conclude, as new bio-based PU products are being developed, the intent for the end of life should be considered, e.g., designing for recycling/biodegradation, or recovery of block molecules, to facilitate the move toward circular economy principles. Therefore, more research is needed on methods for end-of-life scenarios and associated impacts: products may be composed of different layers, possibly with different properties, making separation challenging.

## 9. Application Fields of NIPUs

**Figure 14:**
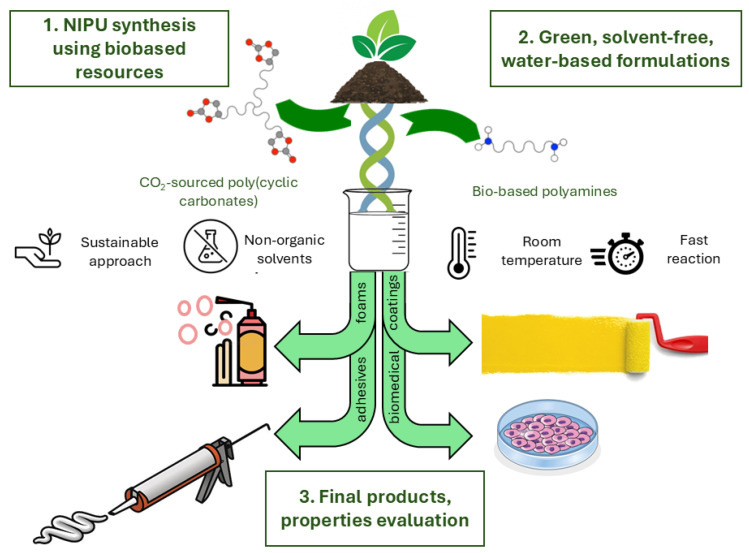
Graphical illustration of various NIPUs’ applications including foams, coatings, adhesives, textiles, and biomedical applications.

Since conventional PUs have manifold applications in the polymer industry, NIPUs can also find place in a wide range of applications. Owing to their properties, they are slowly emerging for various applications, mainly in structural domains where NIPUs are used as coatings, adhesives, and foams supplementing durability and high performance. NIPUs are a step towards safer, more sustainable materials and the pathways to innovations that might answer such industrial and societal challenges. In addition, NIPUs are being explored for biomedical applications, including drug delivery systems, tissue engineering, and prosthetic parts, due to their biocompatibility and tunable mechanical properties (Figure 14).

The variety of possible applications of NIPUs is likely to be expanded as a result of the increasing interest in both industrial and academic research. Some of these application fields are further examined in this section.

### 9.1. NIPUs for Coatings

**Figure 15:**
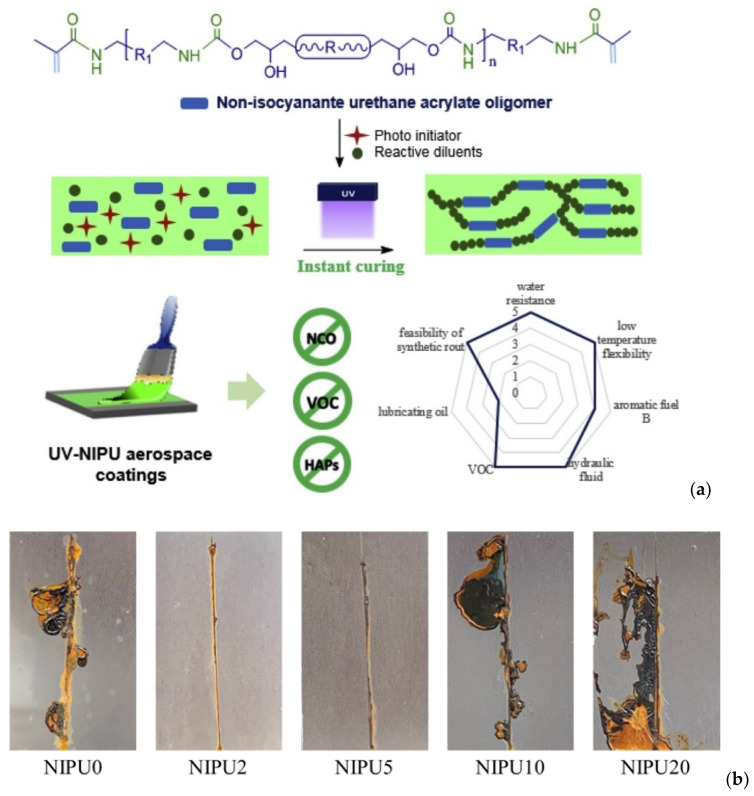
(**a**) NIPUs application in radiation-curable aerospace coatings [125] and (**b**) NIPU anti-corrosion activity [129].

The interest in NIPU coatings is based on their versatility, which leads to a great variety of properties, including soft/flexible and hard/rigid final materials, that can exhibit excellent wetting on metals, alloys, and wood surfaces. Adapting the synthetic route is possible by a careful selection of the main reactants (carbonates, amines, chain extenders, etc.), but also through the incorporation of different additives (pigments, thickeners, surfactants, etc.) that can contribute to the development of functional coatings [121,122]. Their excellent characteristics include their non-porous structure, which helps them exhibit long-term corrosion protection against the corrosive media and provides robustness compared to PU coatings (Figure 15a) [123]. Their enhanced chemical resistance mainly derives from their structure, as the intra- and intermolecular hydrogen bonds of those molecules favor the formation of strong bonds [124]. As a result, NIPU coatings can satisfy a wide range of applications, including industrial, decorative, and other types of monolithic coatings, as well as microelectronics and photonics [125].

Various studies concerning the use of NIPUs for coating applications have already been published. In a study by Liu et al. [126], the preparation of a series of novel biomass-based NIPU coatings was successfully achieved using gallic acid-based cyclic carbonate with diamines. The deriving NIPUs were modified with epoxy-functionalized polyhedral oligomeric silsesquioxanes (POSSs) to form NIPU/POSS coatings with chemically linked POSS groups. The coatings showed excellent impact strength, flexibility, adhesion, thermal stability, and pencil hardness, but suffered from low water resistance. By introducing POSSs to the NIPU networks, the pencil hardness, thermal stability, and water resistance were enhanced, while the adhesion of the NIPU/POSS coatings was observed to be slightly decreased.

Following the same concept and aiming to further improve the chemical resistance of NIPUs, Wu et al. [67] introduced two kinds of diglycidyl ethers, bisphenol AF and perfluorooctyl glycidyl ether. They synthesized two different cyclic carbonates, bisphenol AF and perfluorooctyl cyclic carbonates, that were effectively used to prepare fluorine-containing NIPU coatings. The effects of perfluorooctyl cyclic carbonate amounts on the properties of NIPU were investigated, and the produced NIPU coatings exhibited excellent impact resistance, adhesion, and flexibility, as well as thermal stability. Additionally, the coatings possessed modest water uptake, corrosion resistance properties, and exceptional oleophobic/hydrophobic properties.

In accordance with green chemistry principles, the study by Ling and Zhou [127] explored the utilization of linseed oil-based cyclic carbonate for the preparation of fast-curing waterborne NIPUs for application in coatings. Different formulations of those linseed oil-based NIPU coatings were designed by modifying the urethane content and internal dispersion agent content. The deriving of NIPU coatings with various formulations achieved a broad range of thermal stabilities, viscoelastic properties, and mechanical properties. The remarkable results of their study showed that linseed oil-based waterborne NIPU coatings exhibited characteristics comparable to both a solvent-borne NIPU coating and a commercial waterborne traditional isocyanate-based polyurethane coating.

Among other interesting properties, NIPUs seem to present enhanced anti-smudge capability and excellent mechanical properties. For instance, the work of Lui et al. [128] describes the development of novel NIPU coating systems derived from pentaerythritol glycidyl ether-based cyclic carbonate, single-end cyclic carbonate polydimethysiloxane, and ethylene imine polymer. The coatings exhibited striking liquid repellency against a wide variety of liquids including oils, water, milk, and cola. Additionally, the scientists observed that dust could slide down with water, and there are no remaining dust and water traces on the coated surface, indicating that the NIPU coatings demonstrated exceptional self-cleaning performance. Furthermore, these coatings exhibited remarkable inhibition of ink deposition, maintaining anti-ink performance after thousands of abrasion cycles, indicating that NIPU coatings possessed practical application availability.

Another interesting property one can find in NIPUs is their anti-corrosion ability. A series of anti-corrosion NIPU coatings were synthesized by amine-terminated NIPU, tetraethyl orthosilicate, and bisphenol A epoxy by the scientific team of Zhang et al. [129]. In more detail, the amine-terminated NIPU was synthesized from BPA cyclic carbonate and fatty acid amine and, next, the NIPU/TEOS hybrid coatings were formulated with different amounts of tetraethyl orthosilicate. The anti-corrosion performance of the NIPU coatings (Figure 15b) was evaluated by both salt spray and electrochemical impedance spectroscopy. This study revealed the effect of tetraethyl orthosilicate on the anti-corrosion NIPUs.

NIPUs have also been prepared via photocross-linking of mixtures of an acrylate-terminated NIPU oligomer (prepared by a transurethane polycondensation pathway followed by an acrylation reaction). The obtained NIPU materials exhibited superior thermal stability above 255 °C and enhanced mechanical properties [130].

### 9.2. NIPUs for Adhesives

**Figure 16:**
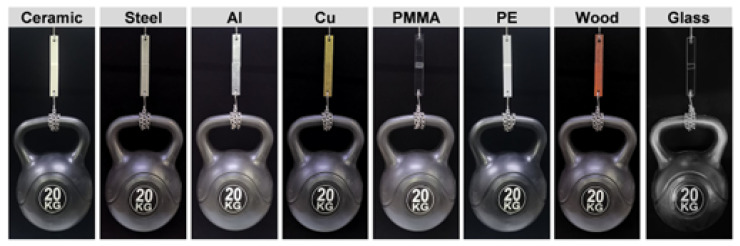
Comparison of NIPUs bonded to various substrates to lift a 20 kg kettlebell [8].

Adhesives’ everyday use is largely expanded from automotive, woodworking, footwear, etc. The adhesives available in the market are usually prepared from polyurethanes, cyanoacrylate, and epoxy resins. PU-based adhesives are being extensively used owing to their excellent flexibility and adhesion (Figure 16) [131].

Adhesives can be prepared via the polyaddition route starting from CO_2_-sourced tricyclic carbonate, with hexamethylene diamine and a catecholamine. Following this process, the team of Panchireddy et al. [132] demonstrated that glues produced by catechol-bearing polyhydroxyurethanes are competing along with the adhesion of commercially available PU glues. Xi et al. [133] developed sucrose-based NIPUs using dimethyl carbonate, hexamethylene diamine, and a silane coupling agent. The NIPUs were tested as adhesives for bonding particleboard. The thermomechanical analysis and DSC revealed that the prepared adhesives performed excellently at 230 °C and that the silane coupling agent promoted significantly the reduction in the curing temperature of the adhesive and allowed it to obtain good bonding at a lower temperature. NIPU adhesives were reported for the first time being used for wood bonding. In their work, Zhang et al. reported the synthesis of ultrastrong NIPU adhesive, which was tested under extreme conditions [134].

Chen et al. [135] utilized soy protein to prepare NIPU thermosetting adhesives for wood panels through a reaction of hexamethylene diamine and dimethyl carbonate. Both linear and branched oligomers were obtained, implying how such oligomeric structures could further cross-link to form a hard, stiff network. The prepared NIPU adhesives seemed to meet the requirements for dry strength and appeared to be suitable for plywood panels in interior grade. Even though their initial samples failed the wet test, it was concluded that the addition of 15% glycerol diglycidyl ether improved the results (still not enough to satisfy the standards). NIPU adhesives based on organosolv lignin were successfully prepared and tested for potential wood applications. Saražin et al. [136] synthesized NIPU adhesives from organosolv lignin, leveraging lignin’s abundance as a raw material. These adhesives displayed satisfactory mechanical properties after hot pressing at 230 °C, an industrially relevant temperature. Adding a silane coupling agent increased their reactivity, allowing effective performance at hot-pressing temperatures below 200 °C.

Bio-derived glycerol diglycidyl ether has been used by Chen et al. [137] as an enhancer to reduce the curing temperature and enhance the bonding performance, while lowering the emission of unsafe substances, such as hexamethylenediamine, in the synthesis process of tannin-based NIPU adhesives. This modification strategy enhanced the bonding properties, reduced the energy consumption during preparation, and decreased the release of dangerous substances, according to the guidelines for a cleaner production process. NIPU adhesives have rapidly become a vital area of interest within the scientific research community. Zhang et al. [138] synthesized bio-based NIPU adhesives using cashew phenol cyclic carbonate. In more detail, they succeeded in synthesizing NIPU by the addition route with cashew phenol glycidyl ether and carbon dioxide, followed by curing with a diamine extracted from biomass-derived oils to produce bio-based NIPU for adhesives at room temperature. These NIPUs demonstrated outstanding thermal stability and remarkable adhesion to a great range of substrate materials and showcased enhanced bonding efficacy at very low temperatures (7.78 MPa at −37 °C).

Non-furanic, commercially available humins were employed to synthesize NIPU resins for wood panel adhesives applications. Both neat humin-based NIPUs and tannin-humin NIPUs were synthesized, with the latter outperforming the former in internal bond strength tests. Both formulations met the standards for interior-grade panels [139]. The preparation of NIPUs via polyaddition of (poly)cyclic carbonates is an optimistic alternative for replacing conventional PUs. For this purpose, Gomez-Lopez et al. suggested the combination of dopamine (adhesive promoter) and aminopropyl trimethoxysilane (fast-curing promoter), with trifunctional 5-membered cyclic carbonate, an aromatic diamine, and a hard monomer, aiming to initiate a synergetic effect on the adhesive and rheological properties of the NIPUs. The results demonstrated that the incorporation of both dopamine and aminopropyl trimethoxysilane to NIPUs strongly enhanced the adhesion to different substrates, showing improved performance with both wood and metal than for polymers such as polyamides, high-density polyethylene, etc. [140].

### 9.3. NIPUs for Foams

PU foams are necessary for everyday life applications, including construction, automotive, packaging, furniture, mattresses thermal insulation, shock absorption, sealants, etc. It is expected that global PU foam production will reach USD ~73.57 billion by 2030 [141]. Among the great advantages of PU foams are their lightweight nature, exceptional insulation properties, and durability. Recently, to address PU drawbacks associated with isocyanates, ongoing study, and growth efforts are being pursued, aiming to synthesize environment-friendly and cost-effective NIPU foams utilizing more sustainable resources [68,142,143,144].

In this context, many scientists have conducted studies on self-blown NIPU foams by reacting bio-based resources with a variety of acids as a blowing agent [145]. For instance, Xi et al. [146] reported the synthesis of a glucose-based NIPU rigid foam using glucose, hexamethylenediamine, and dimethyl carbonate. The scientists prepared the glucose-based NIPUs at room temperature using maleic acid (initiator) and glutaraldehyde (cross-linker), opposing the general requirements for high foaming temperatures. The results obtained from the study indicated that self-blown rigid foams possessed enhanced compression and were directly proportional to foam density. The density of the prepared foams thickens reducing maleic acid and increasing glutaraldehyde, while the foams showed poor fire resistance suggesting that the addition of a fire retardant would be essential. More recently, Yang et al. [147] studied the influence of different acids as catalysts for glucose-based NIPUs. Compared to the others, the foam prepared with phosphoric acid presented the best fire resistance with a LOI value of 24.3%, indicating that it possesses a good level of flame retardancy and slightly improved thermal stability due to the flame-retardant effect of the phosphate ion.

Other than that, tannin-based NIPUs have also been widely studied for the preparation of flexible and rigid foams that could potentially replace conventional PUs [148,149]. A thermally and flame-resistant rigid NIPU was produced by Grunlan et al. from tannic acid and chitosan, requiring no catalyst or solvents. A foamed structure was obtained by the addition of glutaraldehyde and four different carboxylic acids: malic acid, maleic acid, citric acid, and aconitic acid. The properties vary with each carboxylic acid used, but in each case, peak thermal degradation and peak heat release are postponed by >100 °C compared to commercial rigid PU foam and a 75% reduction in afterburn time. This bio-based polyurethane eliminates the hazards of traditional PUs while imparting inherent thermal stability and flame resistance uncharacteristic of conventional foams [150].

By the same token, a promising process to design NIPU foams has been developed by utilizing CO_2_ foaming technology [103,151]. Lim et al. [152] investigated CO_2_’s ability to synthesize cyclic carbonates that are used in the preparation of NIPUs by melt step-growth polymerization with a biosourced amino-telechelic oligoamide. In this study, CO_2_ acts both as a foaming agent and a comonomer to obtain low-density foams that offer low thermal conductivity and have an impressive potential for use in insulating materials. Such foams will contribute to energy conservation and savings by reducing CO_2_ emissions. Another noteworthy study by Detrembleur et al. [153] capitalized on the divergent chemistries of amines with cyclic carbonates, creating a polymer network, and thiolactone, delivering in situ a thiol that generates the blowing agent (CO_2_) by reaction with a cyclic carbonate. This one-pot methodology furnishes flexible to rigid foams with open-cell morphology at moderate temperatures. The foams were easily repurposed into films or structural composites by thermal treatment, showing the first example of recyclable NIPU foams.

### 9.4. NIPUs for Biomedical Applications

Nowadays, smart biomaterials are synthesized to mimic tissue behaviors as they display improved properties, including proliferation and cell adhesion. Among them, biocompatible PUs find a wide range of applications in the biomedical field and can be found under the commercial names Tecoflex™ [154], Pellethane^®^ [155], Carbothane™ [156] (Lubrizol, Wickliffe, OH, USA), Bionate^®^ [157], and Carbosil^®^ [158] (DSM, Maastricht, Holland). These fossil-based PUs mirror the wealthy market since they have established a well-trusted choice among scientists’ and surgeons’ communities. However, PUs often fail to meet the strict requirements in terms of cytotoxicity, as well as carcinogenicity and hemocompatibility. These requests usually depend on the aimed living tissue and the estimated contact time with it and are described in different norms, like ISO 10993 [159] (biological evaluation of medical devices), which is currently the international standard for biocompatibility evaluation [159]. The degradation of isocyanates generates mutagenic and carcinogenic diamines, adding to the hazardous nature of PUs. Thus, to avoid the use of toxic isocyanate, NIPU-based biomaterials have gained great interest, owing to their excellent characteristics, such as non-toxicity, sustainability, biocompatibility, and environmentally friendly nature.

**Figure 17:**
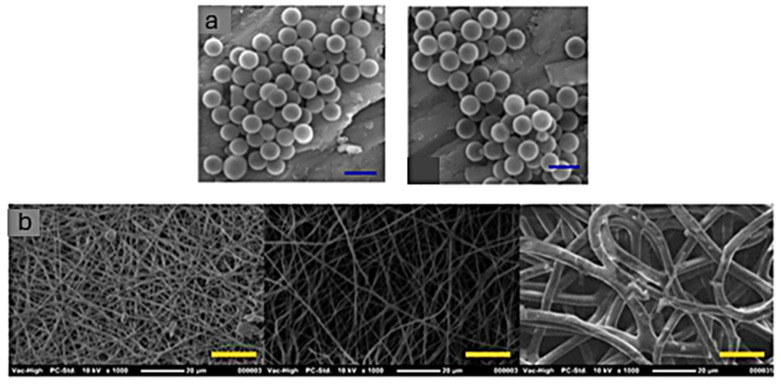
(**a**) Scanning electron microscopy images of the prepared NIPU nanocapsules [9], (**b**) SEM images of NIPU electrospun fibers [10].

In this target, the team of Detrembleur et al. [160] reported for the first time, a one-step one-pot copolymerization reaction, between polyethylene glycol bi-cyclic carbonate and diamines, in the presence of carbonated soybean oil as a cross-linker, in order to obtain NIPU hydrogels in water. Additionally, the study by Pramanik et al. [161] led to the development of mitochondria targeting NIPU nanocapsules (Figure 17a) with a repetitive ester group in their polymeric backbone, with subsequent enzyme-triggered drug release of the bioactive drug. They developed biodegradable, non-isocyanate polyurethane (NIPU) nanocapsules with ester-linked polymeric backbones that degrade in response to esterase enzymes, allowing controlled release of encapsulated bioactive molecules.

Electrospun fiber NIPUs have been synthesized by several research groups, including those led by Aduba and Visser, who developed electrospun fibers from plant-based NIPUs and high molecular weight polycarbonate-based NIPUs, respectively, with both materials aimed at biomedical applications (Figure 17b) [162,163].

Aiming to provide patients with personalized and precise treatment, NIPUs have been efficiently explored as potential manufacturing materials for prosthetic parts. For instance, Melo et al. [164] prepared prosthetic heart valves using PHUs, which were tested in vitro. The produced PHU elastomers were reinforced using a polyester mesh and presented enhanced mechanical properties, equivalent to native valve leaflets. The NIPUs were not responsible for hemolysis and test results indicated low thrombogenicity. In addition, NIPU elastomers with various stiffness values, designed by the functionalization of the hydroxyl groups of a poly-(propylene glycol), were explored for 3D printing using digital light processing. In vitro biocompatibility tests verified the non-cytotoxicity of these materials for human fibroblasts. Moreover, in vitro hemocompatibility tests further revealed that they do not induce hemolytic effects, they do not increase platelet adhesion, nor activate coagulation, highlighting their potential for future applications in the cardiovascular field [165]. Wang et al. [166], in their study, fabricated a 3D-printed NIPU acrylate resin that was photosensitive and possessed superior biocompatibility. NIPU was synthesized via ROP, followed by a ring acrylation reaction. The acrylate NIPU exhibited high tensile and flexural strengths, hemocompatibility, thermal stability, and superior biocompatibility to muscle cells and bone cells, suggesting its potential application in the 3D printing of personalized orthopedic surgical guides.

**Figure 18:**
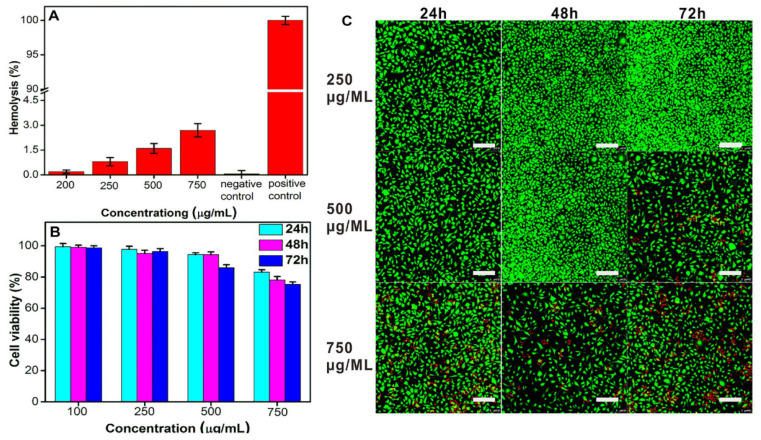
(**A**) Hemolysis test: NIPU shows minimal hemolysis (2.8%) even at 750 μg/mL, indicating it is non-toxic to RBCs. (**B**) Cell viability: The MTT assay confirms over 95% viability at 100–250 μg/mL across 24–72 h. Viability remains above 85% at 500 μg/mL and slightly decreases to 75% at 750 μg/mL, demonstrating excellent biocompatibility with L929 fibroblasts. (**C**) Cytotoxicity (CLSM): Alive (green) and dead (red) cell staining shows predominant green fluorescence at 250–500 μg/mL after 72 h [167].

Furthermore, the thermoresponsive properties of NIPUs have also garnered attention. For instance, one research team reported a reversible thermoresponsive, hemocompatible, and biocompatible NIPU, along with a UV- and thermo-responsive polyurethane-acrylate-based NIPU. These innovations highlight the potential of NIPUs for use in biomedical devices [167,168]. For instance, the biocompatibility and hemocompatibility results of the work of Zhao et al., as shown in Figure 18, verify that NIPHU exhibits very low toxicity with an excellent potentiality towards biomaterials.

Further, the application of NIPUs in medical dressings has been explored, especially in wound care, where NIPUs with antimicrobial properties are shown to control infection and promote healing [169]. Biocompatible NIPUs have been considered suitable for 3D printing, enabling the production of tailored wound-healing films and other biomedical devices [170,171].

### 9.5. Additional NIPU Applications

Recently, Blattmann et al. [142] employed rigid cyclic carbonates (i.e., carbonated trimethylolpropane glycidylether (TMPGC)) that were blended with the corresponding flexible cyclic carbonate (i.e., ethoxylated TMPGC (EO-TMPGC)), which lowers monomer viscosity and reactivity. The resulting NIPU foams were rendered flexible and soft, as verified by simultaneously declining storage modulus and Tg. The flexible NIPU foams exhibited low density, enhanced mechanical hysteresis, and tailored hardness, meeting the required demands of various applications like automotive seating.

A series of sustainable and UV-curable NIPU acrylate (NIPU-AC) oligomers, with different structures and acrylate equivalent weights, were synthesized and tested by the scientific group of Zareanshahraki et al. [125] as a primary building block of UV-curable coatings for aerospace applications. Synthesis of the NIPU-AC oligomers was carried out in three steps. Test analysis results showed that the products that were developed meet the aerospace criteria in terms of performance properties, such as low-temperature flexibility and resistance to specific chemicals/fluids.

The unique properties of NIPUs make them an attractive option for developing sustainable and eco-friendly textile products that meet the growing consumer demand for responsibly produced goods. Mao et al. [4] developed a sustainable non-isocyanate polyurethane with a cross-linked network (CNIPU) using glycerol carbonate, polyamine, and diglycidyl ether via a non-isocyanate route. CNIPU can be used as a binder in cotton pigment dyeing, demonstrating eco-friendly potential and offering a green solution for sustainable textiles.

The potential application of NIPUs in agriculture is in the development of biodegradable coatings for seeds and fertilizers [3,172]. These coatings can protect seeds and fertilizers from moisture, UV light, and pests while also providing a slow-release mechanism for fertilizers. NIPU coatings have been shown to have good adhesion to substrates and sound barrier and mechanical properties, making them a promising alternative to traditional coatings. One notable advantage of waste-derived NIPUs is their reduced carbon footprint, as they are produced from renewable and sustainable resources [3,172].

## 10. Challenges and Opportunities in Scaling Up NIPU Production

It is significant to understand how shifts from manufacturing conventional PUs to manufacturing NIPUs may influence their cost and sustainability. NIPUs face several limitations and challenges that hinder their widespread adoption as replacements for traditional PUs. One major difficulty is achieving the same level of mechanical strength, elasticity, and chemical resistance offered by conventional PUs, which are optimized through the use of highly reactive but toxic isocyanates. The slower polymerization kinetics of NIPU synthesis, extended reaction time, and the necessity for specific catalysts or reaction conditions can complicate production and increase costs.

Up to now, the industrial production of NIPUs has not been thoroughly evaluated for its cost competitiveness. In a techno-economic analysis conducted by Liang and his group [113], it was underlined that raw materials, specifically amines, drive the total NIPU production costs, leading to increased expenses compared to traditional PUs. They calculated the minimum selling prices (MSPs) of PHU and NIPU at 5.37 and 7.16 USD/kg, respectively, which are higher than the baseline MSPs of traditional PU flexible foams, ranging from 3.53 to 4.49 USD/kg. However, with process improvements, the MSPs of PHU and NIPTU could be reduced to 3.15 and 4.39 USD/kg, respectively.

Limited scalability and availability of suitable bio-based raw materials, such as cyclic carbonates and diamines, pose supply chain issues. For example, soybean oil (SBO) is generally considered a relatively affordable and abundant bio-based raw material, making it an attractive option for sustainable industrial applications. To date, SBO is the second largest source of vegetable oil after palm oil [38]. With a market price of 0.93 EUR/kg, SBO is not inherently expensive for NIPU production. However, valid concerns and criticisms have been raised regarding their potential effect on food security [173]. Momodu et al. [174] studied the feasibility of NIPU production in Nigeria, claiming that it still faces several challenges, including the need for large reactors due to long reaction times, which can impact annual production output.

Generally, NIPU technology has not yet attained the stage of industrial implementation, even though it is promising. Addressing present challenges includes a thorough evaluation of various factors like the selection of monomer ratio, solvent, catalyst, and reaction parameters, which collectively contribute to enhancing NIPU properties. Additionally, broader challenges like scalability and cost-effectiveness must be addressed for NIPUs to be accepted as a suitable alternative to the benchmark PUs. To achieve this, the development of a scalable synthetic route and the implementation of comprehensive cycle assessments are essential steps that will contribute to the competitiveness of NIPUs.

## 11. Conclusions

To conclude, NIPUs are a promising class of sustainable polymers that address both environmental and health concerns connected with conventional PUs. However, their properties may not yet completely correspond to those of PUs in all uses. More specifically, the mechanical strength, elasticity, and thermal resistance of NIPUs require additional optimization so that they are at least equal to, or preferably better than, PUs. Notwithstanding these barriers, the research conducted so far has begun to address these problems, and for some applications, NIPUs have proven to be effective substitutes. Nonetheless, the current production costs, which are frequently increased by the use of specialized monomers or processes, remain an obstacle to their universal use. Future studies should revolve around growing cost-effective and scalable techniques, developing NIPU compatibility against commercial materials, and utilizing alternative bio-based resources. Moreover, studies on the performance and longevity of further tested NIPUs under industrial and other high-stress conditions will provide a better foundation for them to be adopted as a more sustainable approach.
